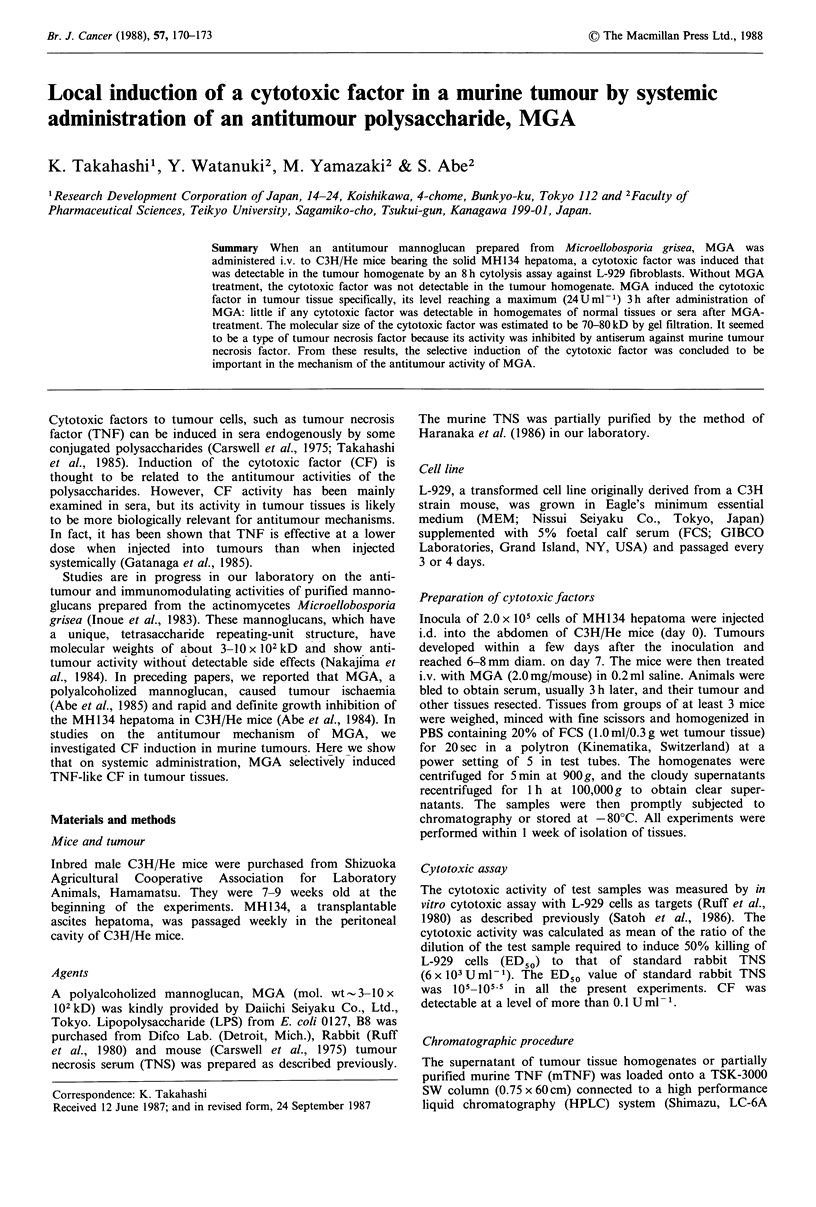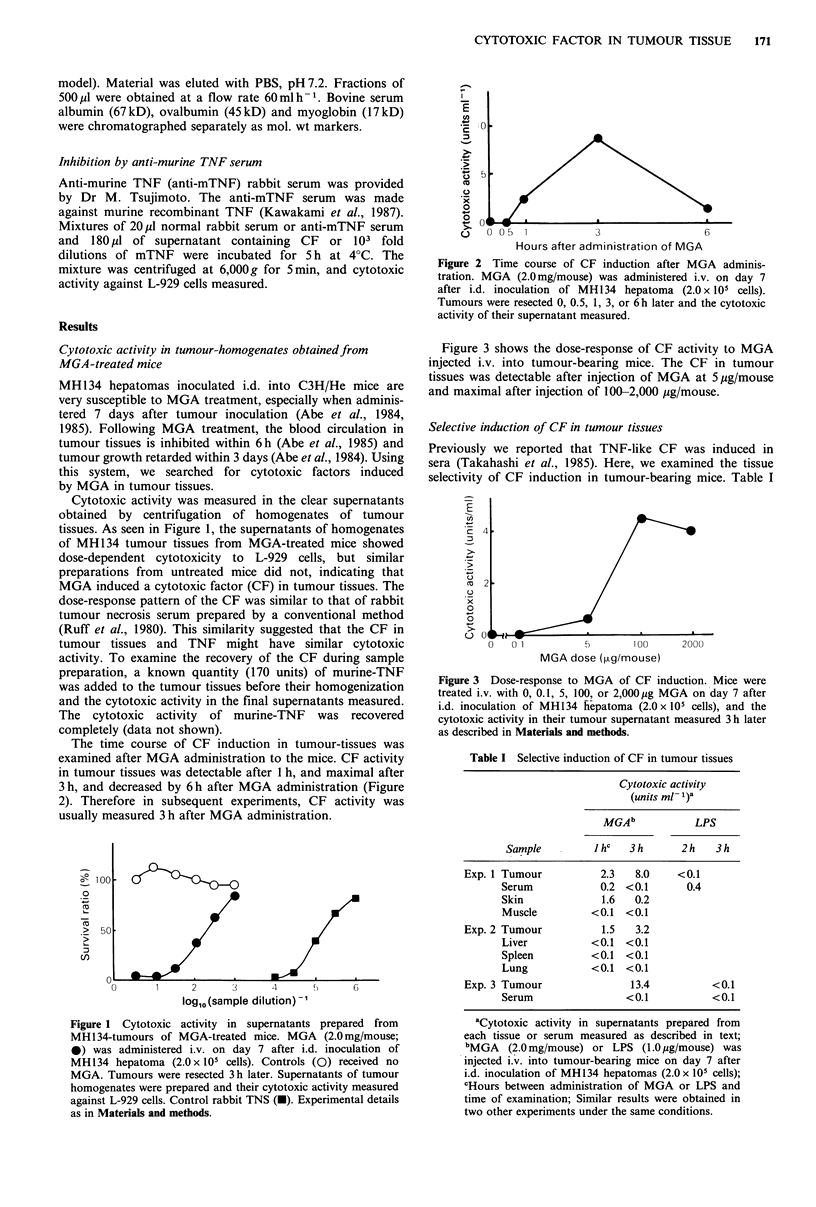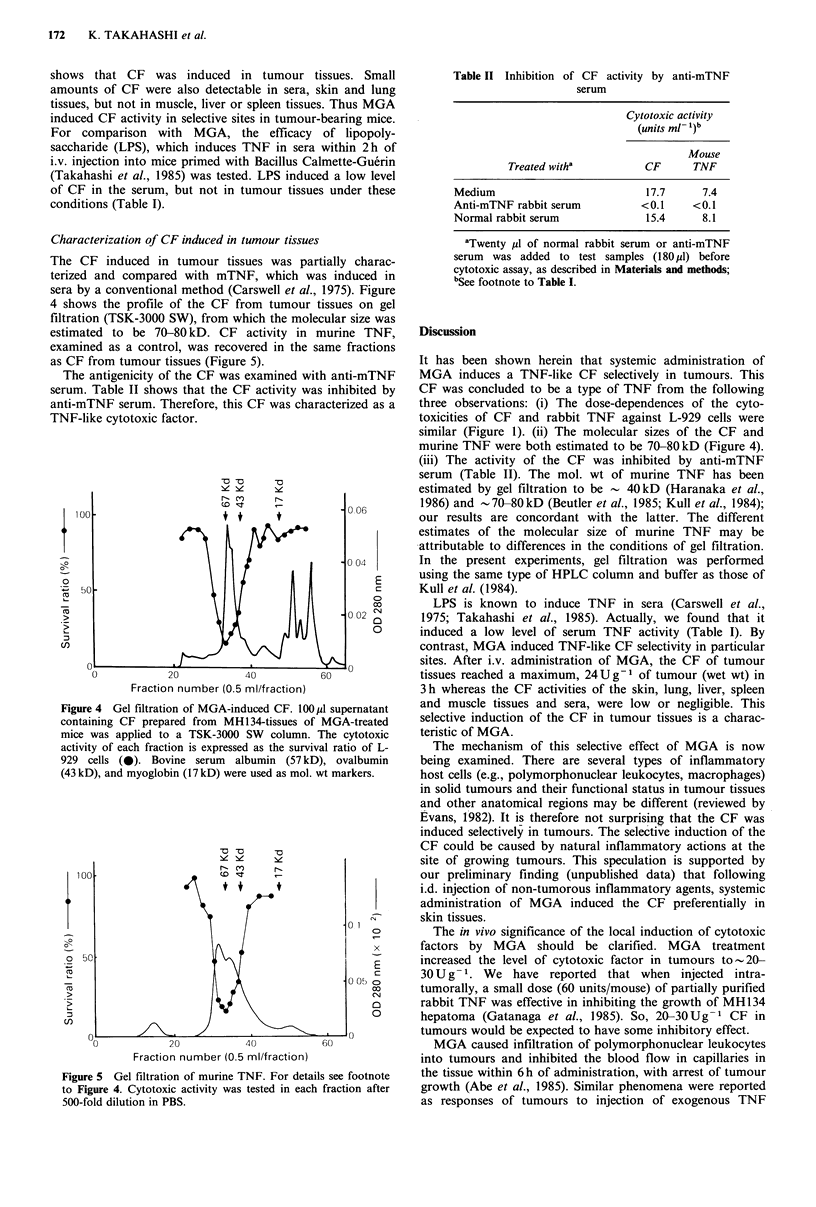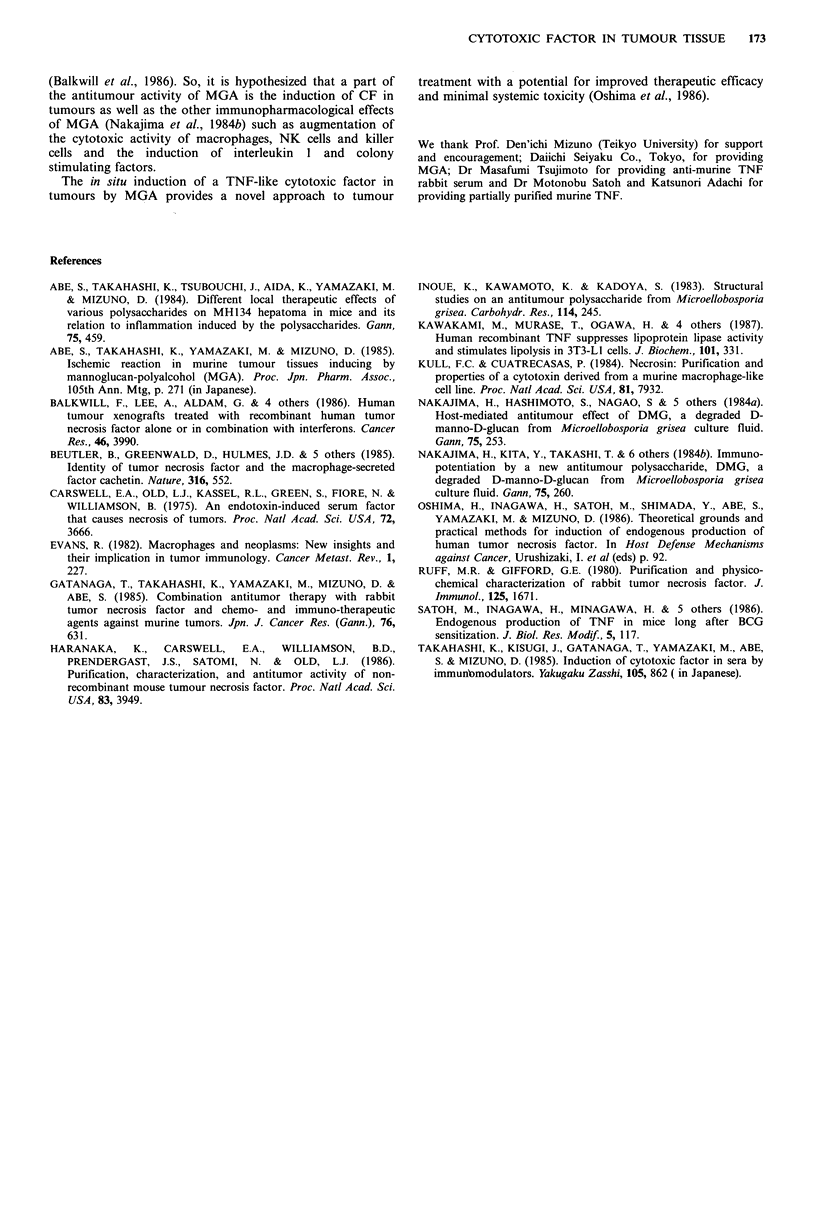# Local induction of a cytotoxic factor in a murine tumour by systemic administration of an antitumour polysaccharide, MGA.

**DOI:** 10.1038/bjc.1988.35

**Published:** 1988-02

**Authors:** K. Takahashi, Y. Watanuki, M. Yamazaki, S. Abe

**Affiliations:** Research Development Corporation of Japan, Tokyo.

## Abstract

When an antitumour mannoglucan prepared from Microellobosporia grisea, MGA was administered i.v. to C3H/He mice bearing the solid MH134 hepatoma, a cytotoxic factor was induced that was detectable in the tumour homogenate by an 8 h cytolysis assay against L-929 fibroblasts. Without MGA treatment, the cytotoxic factor was not detectable in the tumour homogenate. MGA induced the cytotoxic factor in tumour tissue specifically, its level reaching a maximum (24 U ml-1) 3 h after administration of MGA: little if any cytotoxic factor was detectable in homogenates of normal tissues or sera after MGA-treatment. The molecular size of the cytotoxic factor was estimated to be 70-80 kD by gel filtration. It seemed to be a type of tumour necrosis factor because its activity was inhibited by antiserum against murine tumour necrosis factor. From these results, the selective induction of the cytotoxic factor was concluded to be important in the mechanism of the antitumour activity of MGA.


© The Macmillan Press Ltd., 1988

### Local induction of a cytotoxic factor in a murine tumour by systemic administration of an antitumour polysaccharide, MGA

K. Takahashi[1], Y. Watanuki[2], M. Yamazaki[2] & S. Abe[2]

[1]*Research Development Corporation of Japan, 14–24, Koishikawa, 4-chome, Bunkyo-ku, Tokyo 112 and* [2]*Faculty of Pharmaceutical Sciences, Teikyo University, Sagamiko-cho, Tsukui-gun, Kanagawa 199-01, Japan.*

**Summary** When an antitumour mannoglucan prepared from *Microellobosporia grisea*, MGA was administered i.v. to C3H/He mice bearing the solid MH134 hepatoma, a cytotoxic factor was induced that was detectable in the tumour homogenate by an 8 h cytolysis assay against L-929 fibroblasts. Without MGA treatment, the cytotoxic factor was not detectable in the tumour homogenate. MGA induced the cytotoxic factor in tumour tissue specifically, its level reaching a maximum ($24\,U\,ml^{-1}$) 3 h after administration of MGA: little if any cytotoxic factor was detectable in homogemates of normal tissues or sera after MGA-treatment. The molecular size of the cytotoxic factor was estimated to be 70–80 kD by gel filtration. It seemed to be a type of tumour necrosis factor because its activity was inhibited by antiserum against murine tumour necrosis factor. From these results, the selective induction of the cytotoxic factor was concluded to be important in the mechanism of the antitumour activity of MGA.

Cytotoxic factors to tumour cells, such as tumour necrosis factor (TNF) can be induced in sera endogenously by some conjugated polysaccharides (Carswell *et al.*, 1975; Takahashi *et al.*, 1985). Induction of the cytotoxic factor (CF) is thought to be related to the antitumour activities of the polysaccharides. However, CF activity has been mainly examined in sera, but its activity in tumour tissues is likely to be more biologically relevant for antitumour mechanisms. In fact, it has been shown that TNF is effective at a lower dose when injected into tumours than when injected systemically (Gatanaga *et al.*, 1985).

Studies are in progress in our laboratory on the antitumour and immunomodulating activities of purified mannoglucans prepared from the actinomycetes *Microellobosporia grisea* (Inoue *et al.*, 1983). These mannoglucans, which have a unique, tetrasaccharide repeating-unit structure, have molecular weights of about $3–10\times10^2$ kD and show antitumour activity without detectable side effects (Nakajima *et al.*, 1984). In preceding papers, we reported that MGA, a polyalcoholized mannoglucan, caused tumour ischaemia (Abe *et al.*, 1985) and rapid and definite growth inhibition of the MH134 hepatoma in C3H/He mice (Abe *et al.*, 1984). In studies on the antitumour mechanism of MGA, we investigated CF induction in murine tumours. Here we show that on systemic administration, MGA selectively induced TNF-like CF in tumour tissues.

### Materials and methods

### *Mice and tumour*

Inbred male C3H/He mice were purchased from Shizuoka Agricultural Cooperative Association for Laboratory Animals, Hamamatsu. They were 7–9 weeks old at the beginning of the experiments. MH134, a transplantable ascites hepatoma, was passaged weekly in the peritoneal cavity of C3H/He mice.

### *Agents*

A polyalcoholized mannoglucan, MGA (mol. wt $\sim 3–10\times10^2$ kD) was kindly provided by Daiichi Seiyaku Co., Ltd., Tokyo. Lipopolysaccharide (LPS) from *E. coli* 0127, B8 was purchased from Difco Lab. (Detroit, Mich.), Rabbit (Ruff *et al.*, 1980) and mouse (Carswell *et al.*, 1975) tumour necrosis serum (TNS) was prepared as described previously. The murine TNS was partially purified by the method of Haranaka *et al.* (1986) in our laboratory.

### *Cell line*

L-929, a transformed cell line originally derived from a C3H strain mouse, was grown in Eagle's minimum essential medium (MEM; Nissui Seiyaku Co., Tokyo, Japan) supplemented with 5% foetal calf serum (FCS; GIBCO Laboratories, Grand Island, NY, USA) and passaged every 3 or 4 days.

### *Preparation of cytotoxic factors*

Inocula of $2.0\times10^5$ cells of MH134 hepatoma were injected i.d. into the abdomen of C3H/He mice (day 0). Tumours developed within a few days after the inoculation and reached 6–8 mm diam. on day 7. The mice were then treated i.v. with MGA (2.0 mg/mouse) in 0.2 ml saline. Animals were bled to obtain serum, usually 3 h later, and their tumour and other tissues resected. Tissues from groups of at least 3 mice were weighed, minced with fine scissors and homogenized in PBS containing 20% of FCS (1.0 ml/0.3 g wet tumour tissue) for 20 sec in a polytron (Kinematika, Switzerland) at a power setting of 5 in test tubes. The homogenates were centrifuged for 5 min at 900 *g*, and the cloudy supernatants recentrifuged for 1 h at 100,000 *g* to obtain clear supernatants. The samples were then promptly subjected to chromatography or stored at −80°C. All experiments were performed within 1 week of isolation of tissues.

### *Cytotoxic assay*

The cytotoxic activity of test samples was measured by *in vitro* cytotoxic assay with L-929 cells as targets (Ruff *et al.*, 1980) as described previously (Satoh *et al.*, 1986). The cytotoxic activity was calculated as mean of the ratio of the dilution of the test sample required to induce 50% killing of L-929 cells ($ED_{50}$) to that of standard rabbit TNS ($6\times10^3\,U\,ml^{-1}$). The $ED_{50}$ value of standard rabbit TNS was $10^5$–$10^{5.5}$ in all the present experiments. CF was detectable at a level of more than $0.1\,U\,ml^{-1}$.

### *Chromatographic procedure*

The supernatant of tumour tissue homogenates or partially purified murine TNF (mTNF) was loaded onto a TSK-3000 SW column (0.75 × 60 cm) connected to a high performance liquid chromatography (HPLC) system (Shimazu, LC-6A

Correspondence: K. Takahashi
Received 12 June 1987; and in revised form, 24 September 1987

model). Material was eluted with PBS, pH 7.2. Fractions of 500 $\mu$l were obtained at a flow rate 60 ml $h^{-1}$. Bovine serum albumin (67 kD), ovalbumin (45 kD) and myoglobin (17 kD) were chromatographed separately as mol. wt markers.

*Inhibition by anti-murine TNF serum*

Anti-murine TNF (anti-mTNF) rabbit serum was provided by Dr M. Tsujimoto. The anti-mTNF serum was made against murine recombinant TNF (Kawakami *et al.*, 1987). Mixtures of 20 $\mu$l normal rabbit serum or anti-mTNF serum and 180 $\mu$l of supernatant containing CF or $10^3$ fold dilutions of mTNF were incubated for 5 h at 4°C. The mixture was centrifuged at 6,000 *g* for 5 min, and cytotoxic activity against L-929 cells measured.

### Results

*Cytotoxic activity in tumour-homogenates obtained from MGA-treated mice*

MH134 hepatomas inoculated i.d. into C3H/He mice are very susceptible to MGA treatment, especially when administered 7 days after tumour inoculation (Abe *et al.*, 1984, 1985). Following MGA treatment, the blood circulation in tumour tissues is inhibited within 6 h (Abe *et al.*, 1985) and tumour growth retarded within 3 days (Abe *et al.*, 1984). Using this system, we searched for cytotoxic factors induced by MGA in tumour tissues.

Cytotoxic activity was measured in the clear supernatants obtained by centrifugation of homogenates of tumour tissues. As seen in Figure 1, the supernatants of homogenates of MH134 tumour tissues from MGA-treated mice showed dose-dependent cytotoxicity to L-929 cells, but similar preparations from untreated mice did not, indicating that MGA induced a cytotoxic factor (CF) in tumour tissues. The dose-response pattern of the CF was similar to that of rabbit tumour necrosis serum prepared by a conventional method (Ruff *et al.*, 1980). This similarity suggested that the CF in tumour tissues and TNF might have similar cytotoxic activity. To examine the recovery of the CF during sample preparation, a known quantity (170 units) of murine-TNF was added to the tumour tissues before their homogenization and the cytotoxic activity in the final supernatants measured. The cytotoxic activity of murine-TNF was recovered completely (data not shown).

The time course of CF induction in tumour-tissues was examined after MGA administration to the mice. CF activity in tumour tissues was detectable after 1 h, and maximal after 3 h, and decreased by 6 h after MGA administration (Figure 2). Therefore in subsequent experiments, CF activity was usually measured 3 h after MGA administration.

**Figure 1** Cytotoxic activity in supernatants prepared from MH134-tumours of MGA-treated mice. MGA (2.0 mg/mouse; ●) was administered i.v. on day 7 after i.d. inoculation of MH134 hepatoma ($2.0 \times 10^5$ cells). Controls (○) received no MGA. Tumours were resected 3 h later. Supernatants of tumour homogenates were prepared and their cytotoxic activity measured against L-929 cells. Control rabbit TNS (■). Experimental details as in **Materials and methods**.

**Figure 2** Time course of CF induction after MGA administration. MGA (2.0 mg/mouse) was administered i.v. on day 7 after i.d. inoculation of MH134 hepatoma ($2.0 \times 10^5$ cells). Tumours were resected 0, 0.5, 1, 3, or 6 h later and the cytotoxic activity of their supernatant measured.

Figure 3 shows the dose-response of CF activity to MGA injected i.v. into tumour-bearing mice. The CF in tumour tissues was detectable after injection of MGA at 5 $\mu$g/mouse and maximal after injection of 100–2,000 $\mu$g/mouse.

*Selective induction of CF in tumour tissues*

Previously we reported that TNF-like CF was induced in sera (Takahashi *et al.*, 1985). Here, we examined the tissue selectivity of CF induction in tumour-bearing mice. Table I

**Figure 3** Dose-response to MGA of CF induction. Mice were treated i.v. with 0, 0.1, 5, 100, or 2,000 $\mu$g MGA on day 7 after i.d. inoculation of MH134 hepatoma ($2.0 \times 10^5$ cells), and the cytotoxic activity in their tumour supernatant measured 3 h later as described in **Materials and methods**.

**Table I** Selective induction of CF in tumour tissues

| | | Cytotoxic activity (units $ml^{-1}$)[a] | | | |
|---|---|---|---|---|---|
| | | MGA[b] | | LPS | |
| | Sample | 1 h[c] | 3 h | 2 h | 3 h |
| Exp. 1 | Tumour | 2.3 | 8.0 | <0.1 | |
| | Serum | 0.2 | <0.1 | 0.4 | |
| | Skin | 1.6 | 0.2 | | |
| | Muscle | <0.1 | <0.1 | | |
| Exp. 2 | Tumour | 1.5 | 3.2 | | |
| | Liver | <0.1 | <0.1 | | |
| | Spleen | <0.1 | <0.1 | | |
| | Lung | <0.1 | <0.1 | | |
| Exp. 3 | Tumour | | 13.4 | | <0.1 |
| | Serum | | <0.1 | | <0.1 |

[a]Cytotoxic activity in supernatants prepared from each tissue or serum measured as described in text; [b]MGA (2.0 mg/mouse) or LPS (1.0 $\mu$g/mouse) was injected i.v. into tumour-bearing mice on day 7 after i.d. inoculation of MH134 hepatomas ($2.0 \times 10^5$ cells); [c]Hours between administration of MGA or LPS and time of examination; Similar results were obtained in two other experiments under the same conditions.

shows that CF was induced in tumour tissues. Small amounts of CF were also detectable in sera, skin and lung tissues, but not in muscle, liver or spleen tissues. Thus MGA induced CF activity in selective sites in tumour-bearing mice. For comparison with MGA, the efficacy of lipopolysaccharide (LPS), which induces TNF in sera within 2 h of i.v. injection into mice primed with Bacillus Calmette-Guérin (Takahashi *et al.*, 1985) was tested. LPS induced a low level of CF in the serum, but not in tumour tissues under these conditions (Table I).

*Characterization of CF induced in tumour tissues*

The CF induced in tumour tissues was partially characterized and compared with mTNF, which was induced in sera by a conventional method (Carswell *et al.*, 1975). Figure 4 shows the profile of the CF from tumour tissues on gel filtration (TSK-3000 SW), from which the molecular size was estimated to be 70–80 kD. CF activity in murine TNF, examined as a control, was recovered in the same fractions as CF from tumour tissues (Figure 5).

The antigenicity of the CF was examined with anti-mTNF serum. Table II shows that the CF activity was inhibited by anti-mTNF serum. Therefore, this CF was characterized as a TNF-like cytotoxic factor.

**Figure 4** Gel filtration of MGA-induced CF. 100 $\mu$l supernatant containing CF prepared from MH134-tissues of MGA-treated mice was applied to a TSK-3000 SW column. The cytotoxic activity of each fraction is expressed as the survival ratio of L-929 cells (●). Bovine serum albumin (57 kD), ovalbumin (43 kD), and myoglobin (17 kD) were used as mol. wt markers.

**Figure 5** Gel filtration of murine TNF. For details see footnote to **Figure 4**. Cytotoxic activity was tested in each fraction after 500-fold dilution in PBS.

**Table II** Inhibition of CF activity by anti-mTNF serum

| | *Cytotoxic activity (units ml$^{-1}$)*[b] | |
|---|---|---|
| *Treated with*[a] | *CF* | *Mouse TNF* |
| Medium | 17.7 | 7.4 |
| Anti-mTNF rabbit serum | <0.1 | <0.1 |
| Normal rabbit serum | 15.4 | 8.1 |

[a]Twenty $\mu$l of normal rabbit serum or anti-mTNF serum was added to test samples (180 $\mu$l) before cytotoxic assay, as described in **Materials and methods**; [b]See footnote to **Table I**.

### Discussion

It has been shown herein that systemic administration of MGA induces a TNF-like CF selectively in tumours. This CF was concluded to be a type of TNF from the following three observations: (i) The dose-dependences of the cytotoxicities of CF and rabbit TNF against L-929 cells were similar (Figure 1). (ii) The molecular sizes of the CF and murine TNF were both estimated to be 70–80 kD (Figure 4). (iii) The activity of the CF was inhibited by anti-mTNF serum (Table II). The mol. wt of murine TNF has been estimated by gel filtration to be ~ 40 kD (Haranaka *et al.*, 1986) and ~70–80 kD (Beutler *et al.*, 1985; Kull *et al.*, 1984); our results are concordant with the latter. The different estimates of the molecular size of murine TNF may be attributable to differences in the conditions of gel filtration. In the present experiments, gel filtration was performed using the same type of HPLC column and buffer as those of Kull *et al.* (1984).

LPS is known to induce TNF in sera (Carswell *et al.*, 1975; Takahashi *et al.*, 1985). Actually, we found that it induced a low level of serum TNF activity (Table I). By contrast, MGA induced TNF-like CF selectivity in particular sites. After i.v. administration of MGA, the CF of tumour tissues reached a maximum, 24 U g$^{-1}$ of tumour (wet wt) in 3 h whereas the CF activities of the skin, lung, liver, spleen and muscle tissues and sera, were low or negligible. This selective induction of the CF in tumour tissues is a characteristic of MGA.

The mechanism of this selective effect of MGA is now being examined. There are several types of inflammatory host cells (e.g., polymorphonuclear leukocytes, macrophages) in solid tumours and their functional status in tumour tissues and other anatomical regions may be different (reviewed by Evans, 1982). It is therefore not surprising that the CF was induced selectively in tumours. The selective induction of the CF could be caused by natural inflammatory actions at the site of growing tumours. This speculation is supported by our preliminary finding (unpublished data) that following i.d. injection of non-tumorous inflammatory agents, systemic administration of MGA induced the CF preferentially in skin tissues.

The *in vivo* significance of the local induction of cytotoxic factors by MGA should be clarified. MGA treatment increased the level of cytotoxic factor in tumours to ~20–30 U g$^{-1}$. We have reported that when injected intratumorally, a small dose (60 units/mouse) of partially purified rabbit TNF was effective in inhibiting the growth of MH134 hepatoma (Gatanaga *et al.*, 1985). So, 20–30 U g$^{-1}$ CF in tumours would be expected to have some inhibitory effect.

MGA caused infiltration of polymorphonuclear leukocytes into tumours and inhibited the blood flow in capillaries in the tissue within 6 h of administration, with arrest of tumour growth (Abe *et al.*, 1985). Similar phenomena were reported as responses of tumours to injection of exogenous TNF

(Balkwill *et al.*, 1986). So, it is hypothesized that a part of the antitumour activity of MGA is the induction of CF in tumours as well as the other immunopharmacological effects of MGA (Nakajima *et al.*, 1984*b*) such as augmentation of the cytotoxic activity of macrophages, NK cells and killer cells and the induction of interleukin 1 and colony stimulating factors.

The *in situ* induction of a TNF-like cytotoxic factor in tumours by MGA provides a novel approach to tumour treatment with a potential for improved therapeutic efficacy and minimal systemic toxicity (Oshima *et al.*, 1986).

We thank Prof. Den'ichi Mizuno (Teikyo University) for support and encouragement; Daiichi Seiyaku Co., Tokyo, for providing MGA; Dr Masafumi Tsujimoto for providing anti-murine TNF rabbit serum and Dr Motonobu Satoh and Katsunori Adachi for providing partially purified murine TNF.

### References

ABE, S., TAKAHASHI, K., TSUBOUCHI, J., AIDA, K., YAMAZAKI, M. & MIZUNO, D. (1984). Different local therapeutic effects of various polysaccharides on MH134 hepatoma in mice and its relation to inflammation induced by the polysaccharides. *Gann*, **75**, 459.

ABE, S., TAKAHASHI, K., YAMAZAKI, M. & MIZUNO, D. (1985). Ischemic reaction in murine tumour tissues inducing by mannoglucan-polyalcohol (MGA). *Proc. Jpn. Pharm. Assoc.*, 105th Ann. Mtg, p. 271 (in Japanese).

BALKWILL, F., LEE, A., ALDAM, G. & 4 others (1986). Human tumour xenografts treated with recombinant human tumor necrosis factor alone or in combination with interferons. *Cancer Res.*, **46**, 3990.

BEUTLER, B., GREENWALD, D., HULMES, J.D. & 5 others (1985). Identity of tumor necrosis factor and the macrophage-secreted factor cachetin. *Nature*, **316**, 552.

CARSWELL, E.A., OLD, L.J., KASSEL, R.L., GREEN, S., FIORE, N. & WILLIAMSON, B. (1975). An endotoxin-induced serum factor that causes necrosis of tumors. *Proc. Natl Acad. Sci. USA*, **72**, 3666.

EVANS, R. (1982). Macrophages and neoplasms: New insights and their implication in tumor immunology. *Cancer Metast. Rev.*, **1**, 227.

GATANAGA, T., TAKAHASHI, K., YAMAZAKI, M., MIZUNO, D. & ABE, S. (1985). Combination antitumor therapy with rabbit tumor necrosis factor and chemo- and immuno-therapeutic agents against murine tumors. *Jpn. J. Cancer Res. (Gann.)*, **76**, 631.

HARANAKA, K., CARSWELL, E.A., WILLIAMSON, B.D., PRENDERGAST, J.S., SATOMI, N. & OLD, L.J. (1986). Purification, characterization, and antitumor activity of non-recombinant mouse tumour necrosis factor. *Proc. Natl Acad. Sci. USA*, **83**, 3949.

INOUE, K., KAWAMOTO, K. & KADOYA, S. (1983). Structural studies on an antitumour polysaccharide from *Microellobosporia grisea*. *Carbohydr. Res.*, **114**, 245.

KAWAKAMI, M., MURASE, T., OGAWA, H. & 4 others (1987). Human recombinant TNF suppresses lipoprotein lipase activity and stimulates lipolysis in 3T3-L1 cells. *J. Biochem.*, **101**, 331.

KULL, F.C. & CUATRECASAS, P. (1984). Necrosin: Purification and properties of a cytotoxin derived from a murine macrophage-like cell line. *Proc. Natl Acad. Sci. USA*, **81**, 7932.

NAKAJIMA, H., HASHIMOTO, S., NAGAO, S & 5 others (1984*a*). Host-mediated antitumour effect of DMG, a degraded D-manno-D-glucan from *Microellobosporia grisea* culture fluid. *Gann*, **75**, 253.

NAKAJIMA, H., KITA, Y., TAKASHI, T. & 6 others (1984*b*). Immunopotentiation by a new antitumour polysaccharide, DMG, a degraded D-manno-D-glucan from *Microellobosporia grisea* culture fluid. *Gann*, **75**, 260.

OSHIMA, H., INAGAWA, H., SATOH, M., SHIMADA, Y., ABE, S., YAMAZAKI, M. & MIZUNO, D. (1986). Theoretical grounds and practical methods for induction of endogenous production of human tumor necrosis factor. In *Host Defense Mechanisms against Cancer*, Urushizaki, I. *et al* (eds) p. 92.

RUFF, M.R. & GIFFORD, G.E. (1980). Purification and physico-chemical characterization of rabbit tumor necrosis factor. *J. Immunol.*, **125**, 1671.

SATOH, M., INAGAWA, H., MINAGAWA, H. & 5 others (1986). Endogenous production of TNF in mice long after BCG sensitization. *J. Biol. Res. Modif.*, **5**, 117.

TAKAHASHI, K., KISUGI, J., GATANAGA, T., YAMAZAKI, M., ABE, S. & MIZUNO, D. (1985). Induction of cytotoxic factor in sera by immunomodulators. *Yakugaku Zasshi*, **105**, 862 ( in Japanese).